# Ethanol Concentration-Dependent Alterations in Gene Expression During Acute Binge Drinking in the HIV-1 Transgenic Rat

**DOI:** 10.1111/acer.12077

**Published:** 2013-02-15

**Authors:** Sraboni Sarkar, Sulie L Chang

**Affiliations:** Institute of Neuroimmune Pharmacology, Seton Hall UniversitySouth Orange, New Jersey; Department of Biological Sciences, Seton Hall UniversitySouth Orange, New Jersey

**Keywords:** Blood EtOH Concentration, HIV-1, Adh, Cyp2e1

## Abstract

**Background:**

Binge drinking of high ethanol (EtOH) concentration beverages is common among young adults and can be a risk factor for exposure to sexually transmitted diseases, including HIV-1. We used a novel noninfectious HIV-1 transgenic (HIV-1Tg) rat model that mimics HIV-1 patients in terms of altered immune responses and deficits in cognitive learning and memory to investigate EtOH concentration-dependent effects on 48 alcohol-modulated genes during binge EtOH administration.

**Methods:**

HIV-1Tg and control F344 rats were administered water, 8% EtOH, or 52% EtOH by gavage (i.g.) for 3 days (2.0 g/kg/d). Two hours after final treatment, blood, liver, and spleen were collected from each animal. Serum blood EtOH concentration (BEC) was measured, and gene expression in the liver and spleen was determined using a specifically designed PCR array.

**Results:**

The BEC was significantly higher in the 52% EtOH-treated HIV-1Tg rats compared with the 8% EtOH group; however, the BEC was higher in the 8% EtOH-treated control rats compared with the 52% EtOH group. There was no change in expression of the EtOH metabolism-related genes, Adh1, Adh4, and Cyp2e1, in either the 8 or 52% EtOH-treated HIV-1Tg rats, whereas expression of those genes was significantly higher in the liver of the 52% EtOH control rats, but not in the 8% EtOH group. In the HIV-1Tg rats, expression of the GABA_A_, metabotropic glutamate, and dopamine neurotransmitter receptor genes was significantly increased in the spleen of the 52% EtOH group, but not in the 8% EtOH group, whereas no change was observed in those genes in either of the control groups.

**Conclusions:**

Our data indicate that, in the presence of HIV-1 infection, EtOH concentration-dependent binge drinking can have significantly different molecular effects.

Binge alcohol drinking is a common pattern of consuming excessive amounts of alcohol in a short period of time. The National Institute on Alcohol Abuse and Alcoholism (NIAAA) defines binge drinking as consuming more than 4 alcoholic beverages within a period of 2 hours, elevating the blood alcohol concentration (BAC) to more than 0.08 g% (NIAAA, 2012). In the United States, binge drinking is a popular form of alcohol intake in adults, with more than 75% of the alcohol consumed by binging. Moreover, the highest proportion (>50%) of binge drinkers are in the young adult age group of 18 to 21 years of age (CDC, 2010b). Underage drinkers (below 21 years of age) consume 90% of their alcoholic beverages in the form of binge drinking.

Binge drinking has been correlated with a higher risk for automobile accidents, behavioral problems, and also for contracting sexually transmitted diseases (Naimi et al., 2003; Sarkar et al., 2013). In addition, it has also been found to cause neurocognitive impairment, particularly in young adults (Courtney and Polich, 2009).

Alcoholic beverages vary in their alcohol content, designated as alcohol by volume (ABV) or ethanol (EtOH) concentration, ranging from 5 to 8% ABV in beer and 12% in wine, to as high as 40 to 50% in hard liquor (Sarkar et al., 2013). Although the preferred alcoholic beverage in the United States is beer, adolescents and young adults show a higher propensity to binge on high ABV drinks, such as hard liquor (Siegel et al., 2011).

Recently, we reported that there are differential effects of binge drinking with high EtOH concentration solutions in comparison with low EtOH concentration solutions in the spleen of adolescent rats. We found that GABA_A_ receptor α2 subunit gene expression was significantly decreased with the higher EtOH concentration (Liu et al., 2011). We have also shown that only high concentrations of EtOH (>32% ABV) can activate the hypothalamic supraoptic nucleus, particularly the vasopressin neurons that regulate osmoregulation (Chang et al., 1995). In addition, we recently reported a difference in the time course of blood EtOH concentration (BEC) between 2 different EtOH concentration binge treatments (Sarkar et al., 2013). Taken together, these studies indicate that there are differential physiological effects depending on the EtOH concentration consumed and the pattern of drinking.

Alcohol abuse is a persistent problem in the HIV-1 infected population, with about 50% of HIV-1 patients indulging in regular heavy drinking (Baum et al., 2010; Sarkar et al., 2013). Many studies have reported that regular alcohol use by HIV-1 patients not only delays treatment, but also reduces adherence to medication, often leading to further complications (Chander, 2011; Lucas et al., 2002; Samet et al., 1998; Wagner et al., 2001).

Alcohol abuse also increases a person's susceptibility to secondary infections by suppressing immune responses, both innate and adaptive (Baum et al., 2010; Cook, 1998), which are already compromised in HIV-1 patients (Baum et al., 2010; Flora et al., 2005). HIV-1 viral proteins, in particular Tat, can increase the EtOH-mediated impairment of neutrophil function (Prakash et al., 1998), and act synergistically with EtOH to induce secretion of apoptotic factors (Acheampong et al., 2002) and pro-inflammatory cytokines in brain cells (Flora et al., 2005; Lawson et al., 2011; Mayne et al., 2000).

We used the HIV-1 transgenic (HIV-1Tg) rodent model in this study. The HIV-1Tg rat has similar physiological and behavioral characteristics as HIV-1 patients receiving highly active antiretroviral therapy (HAART; Lashomb et al., 2009; Reid et al., 2001; Vigorito et al., 2007). The HIV-1 provirus in these animals contains a functional deletion of the *gag* and *pol* genes, which eliminates replication; however, the rest of the HIV-1 viral genes, including the *tat*, *gp120*, *nef*, *rev*, and *vif*, are expressed (Chang et al., 2007a,b; Peng et al., 2010; Reid et al., 2001).

The BAC or BEC is defined as the EtOH concentration in the blood after the consumed alcoholic beverage has been metabolized. The change in BAC levels depends primarily on the rate of alcohol absorption from the stomach and small intestines and the rate of metabolism in the liver. In addition, the rate of drinking and the genetic expression of alcohol-metabolizing enzymes also influence the BAC (Zakhari, 2006).

Alcohol is absorbed into the blood in the stomach and small intestine and transported to the liver, where different enzymes work to metabolize the alcohol (Edenberg, 2007; Zakhari, 2006). Alcohol dehydrogenase (ADH) oxidizes EtOH to form acetaldehyde, a toxic byproduct, which is promptly converted to acetate by the enzyme, aldehyde dehydrogenase. Acetate is further metabolized in the liver and also in various tissues to form either carbon dioxide and/or acetyl-CoA, which is used in the synthesis of other byproducts, such as lipids and cholesterol (Zakhari, 2006). ADH enzymes are categorized into several subtypes, depending upon their kinetic activity. When a high concentration of EtOH is ingested, ADH1B and class II enzyme, ADH4, are particularly active in metabolizing the alcohol at a faster rate. In addition, another enzyme, cytochrome P450 2E1 (Cyp2e1), also oxidizes EtOH, particularly at high EtOH concentrations (Zakhari, 2006).

Very little information has been obtained about alcohol metabolism in the HIV-1 disease condition. Haorah and colleagues (2004) reported significantly low Cyp2e1 activity in human monocyte-derived macrophages infected with HIV-1. We have shown that, in HIV-1Tg rats, high EtOH concentration binge consumption induces increased expression of the HIV-1 viral protein, Tat, in the brain, liver, and spleen of the animals (Sarkar et al., 2013), indicating that HIV-1Tg rats are sensitive to high concentrations of EtOH.

In this study, we investigated the EtOH concentration-dependent effects of binge drinking in HIV-1Tg rats by examining: (i) end-point BEC levels, and (ii) changes in gene expression in the liver and spleen after a 3-day binge treatment with low (8%) versus high (52%) EtOH concentrations.

## Materials and Methods

### Animals

Male HIV-1Tg and Fisher/NHsd 344 (F344) normal rats were purchased from Harlan Laboratories (Indianapolis, IN). The animals were housed in clear plastic cages in groups of 3 to 4 in a temperature controlled room (21 to 22°C) with a 12-hour light/12-hour dark illumination cycle, with food and water provided ad libitum. The rats were 70 to 75 postnatal days (PD) old at the start of experimentation. The Institutional Animal Care and Use Committee (IACUC) at Seton Hall University, South Orange, NJ approved the experimental protocol.

### Binge EtOH Treatment

Both HIV-1Tg and normal F344 rats were assigned into 1 of the following groups: HIV-1Tg 0% EtOH (water-control, *n* = 4); HIV-1Tg 8% EtOH (*n* = 4); HIV-1Tg 52% EtOH (*n* = 4); F344 0% EtOH (*n* = 4); F344 8% EtOH (*n* = 4); or F344 52% EtOH (*n* = 4). The groups were administered 0% EtOH, 8% EtOH, or 52% EtOH through gavage (i.g.) once a day (7:00 am) for 3 days for a total of 2.0 g/kg/d. Two hours after the final treatment on Day 3, the blood was collected and the liver and spleen were harvested for total RNA extraction.

### Blood EtOH Concentration

Blood was collected 2 hours after the final EtOH treatment on Day 3. Whole blood from each sample was centrifuged (Sorvall RT6000D; Thermoscientific, Asheville, NC) at 4°C for 20 minutes. The supernatant (serum) was collected. The BEC in the serum was measured using an alcohol oxidase-based fluorometric assay kit from BioVision (Mountain View, CA) following the manufacturer's protocol. Briefly, the serum samples were diluted 1:2,000 with assay buffer, and standards were prepared from a pure EtOH standard (provided) by serial dilution. The samples and standards were incubated with the reaction mixture provided by the manufacturer in a 96-well plate at 37°C, protected from light, for 30 minutes.

### Total RNA Isolation and Reverse Transcription

Total RNA was extracted from the spleen and liver with TRIZOL® (Invitrogen, Carlsbad, CA). The total RNA was further purified using an RNeasy mini-kit (Qiagen, Valencia, CA). Reverse transcription (RT) was performed with 400 μg of total RNA from each sample by converting the RNA into cDNA using Moloney murine leukemia virus (MMLV) reverse transcriptase (Invitrogen). The reactions were incubated in a GeneAmp 2400 Thermocycler (Eppendorf, Westbury, NY) for 1 hour at 37°C, followed by 10 minutes at 67°C. Negative controls were not treated with MMLV reverse transcriptase.

### Real-Time PCR Array

Gene expression was quantified using a custom-made rat PCR array kit and RT^2^ SYBR Green Fluorescin qPCR Master Mix (SA Biosciences, Frederick, MD), according to the manufacturer's instructions. The cDNA samples were mixed with the RT^2^ SYBR Green Fluorescin qPCR Master Mix, then 10 μl of the reaction mixture was placed into designated wells in a 384-well custom-designed PCR array plate. This custom PCR array consists of 46 genes that are known to be regulated by EtOH, including chemokines, cytokines, neurotransmitter receptors (γ-aminobutyric acid [GABA], dopaminergic, glutamate), and EtOH metabolism–related genes and 2 housekeeping genes, *actin beta* (actβ) and *tubulin beta 2b* (tubβ2). Real-time PCR was performed using an ABI Prism 7900HT Fast Detection System (Applied Biosystems, Foster, CA). The PCR mix was denatured for 10 minutes at 95°C, followed by 40 cycles for 15 seconds at 95°C, and 1 minute at 60°C.

### Real-Time PCR

PCR analysis of *Adh1*, *Cyp2e1*, *Gabra1*, *Grm2*, and *Drd1a* was performed with the RT^2^ qPCR Primer Assay (Qiagen) and RT^2^ SYBR Green Master Mix using an ABI Prism 7900HT Fast Detection System (Applied Biosystems) according to the manufacturer's instructions. One microliter of cDNA was used in a 25-μl reaction mixture. The thermocycler parameters were 10 minutes at 95°C, followed by 40 cycles for 15 seconds at 95°C, and 1 minute at 60°C. The data were normalized to actβ, which was used as an internal standard.

### Statistical Analysis

#### BEC Data Analysis

Statistical analysis was performed using GraphPad Prism statistical software (GraphPad Software, Inc., La Jolla, CA). Differences among the strains (HIV-1Tg and F344) and treatment groups (0% EtOH, 8% EtOH, and 52% EtOH) were analyzed by a 2-way analysis of variance, followed by a Bonferroni's post hoc test. Significance was determined at *p* < 0.05.

#### PCR Array Data Analysis

The threshold and baseline values were set manually according to the manufacturer's instructions. The cycle threshold (CT) values used for data analysis were from the PCR array data analysis program provided on the manufacturer's website (http://www.sabiosciences.com/pcrarraydataanalysis.php). Expression of each gene was normalized using 2 housekeeping genes as controls. The expression of each gene was calculated using the ΔΔCT method and compared with the expression in the control. A difference was considered significant at *p* < 0.05. Each value was represented as the mean fold of RNA expression compared with the controls from 3 to 4 biological replicates.

#### Real-Time PCR Data Analysis

Statistical analysis was performed using GraphPad Prism statistical software. The expression of each gene was calculated using the ΔΔCT method and compared with the expression in the control group. A difference was considered significant at *p* < 0.05. Each value was represented as the mean fold of RNA expression compared with the controls from 3 to 4 biological replicates.

## Results

### BEC in HIV-1Tg Rats Following a 3-Day Binge Treatment with 8% EtOH Versus 52% EtOH

**Fig. 1:**
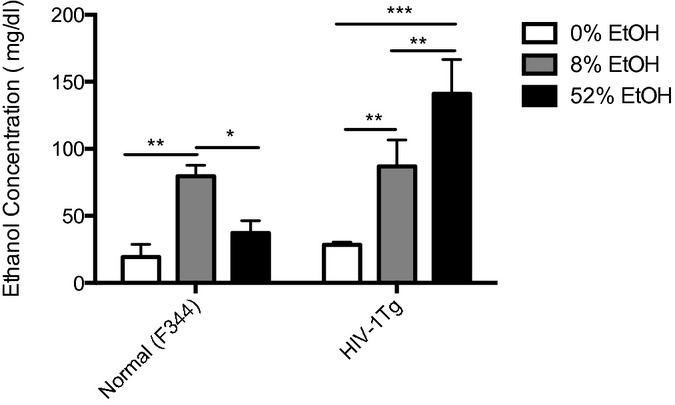
Blood ethanol (EtOH) concentration (BEC) following a 3-day binge treatment with 8% EtOH versus 52% EtOH in HIV-1Tg rats. HIV-1Tg rats were given 0% (water control), 8% EtOH, or 52% EtOH solution (total dose = 2.0 g/kg/d) by gavage (i.g.) for 3 days, and the BEC in serum was determined 2 hours after final treatment. F344 normal rats were used as the control animals. Values represent the mean ± SD (*n* = 3 to 4 rats for each group). *p < 0.05, **p < 0.01, ***p < 0.001.

The BEC was measured in HIV-1Tg and F344 normal rats 2 hours after the final administration of a 3-day binge i.g. treatment with 0% EtOH (control), 8% EtOH, or 52% EtOH solutions (Fig. 1). In the F344 rats, there was a significant increase in BEC in the 8% EtOH group (79.6 ± 4.1 mg/dl) compared with both the 52% EtOH group (37.3 ± 5.3 mg/dl) and the 0% control (19.3 ± 5.4 mg/dl).

In the HIV-1Tg rats, there was a significant increase in BEC in both the 8% EtOH group (87 ± 11.2 mg/dl) and 52% EtOH group (141 ± 12.7 mg/dl) compared with the 0% control (28.4 ± 1.4 mg/dl). In addition, the BEC was significantly increased in the 52% EtOH group compared with the 8% EtOH group.

### EtOH Concentration-Dependent Changes in Gene Expression in the Liver of HIV-1Tg Rats Following a 3-Day Binge Treatment with 8% EtOH Versus 52% EtOH

**Fig. 2:**
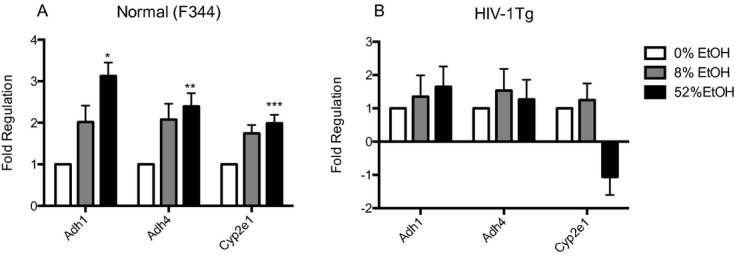
Ethanol (EtOH) concentration-dependent expression of EtOH metabolism-related genes in the liver of HIV-1Tg rats using PCR array analysis. *Adh1*, *Adh4*, and *Cyp2e1* expression was measured in the liver of young adult HIV-1Tg and F344 normal rats treated with 0% EtOH (water control), 8% EtOH, or 52% EtOH in a 3-day binge regimen (total dose of 2.0 g/kg/d), using a custom-designed 48-gene PCR array. The fold change was calculated using the ΔΔCT method relative to the 0% EtOH group. Values represent the mean ± SD (*n* = 3 to 4 rats for each group). *p < 0.05, **p < 0.01, ***p < 0.001.

Using a custom-designed PCR array, we examined changes in gene expression in response to a 3-day binge treatment with 0% (control), 8% EtOH, or 52% EtOH in the liver of HIV-1Tg and F344 normal rats (Fig. 2). In the F344 rats (Fig. 2*A*, Table 1), significant EtOH concentration-dependent changes were observed in the expression of genes associated with EtOH metabolism. Both ADH 1 and 4 (*Adh1* and *Adh4*) were significantly increased in the 52% EtOH group (3.1-fold and 2.4-fold, respectively) compared with the control. There was also an increase in expression in the 8% EtOH group (2-fold *Adh1*; 2.1-fold *Adh4*) compared with control; however, the increase was not statistically significant for *Adh1*. Similarly, *Cyp2e1* gene expression was significantly increased (2-fold) in the 52% EtOH group compared with the control. The increase in the 8% EtOH group (1.7-fold) was not statistically significant.

**Table 1:** Ethanol (EtOH) Concentration-Dependent Expression of EtOH Metabolism-Related Genes in the Liver of HIV-1Tg Rats

	Normal (F344) rats: up-down regulation				HIV-1Tg rats: up-down regulation			
		
Liver	8% EtOH		52% EtOH		8% EtOH		52% EtOH	
				
Gene symbol	Fold regulation	*p*-Value	Fold regulation	*p*-Value	Fold regulation	*p*-Value	Fold regulation	*p*-Value
*Adhl*	2.017	0.121416	3.1279	0.003437	1.352	0.318624	1.6487	0.200644
*Adh4*	2.0781	0.014263	2.3951	0.000577	1.5358	0.170954	1.2677	0.485324
*Cyp2e1*	1.744	0.060158	1.988	0.000085	1.2488	0.306874	−1.0609	0.9217

Significance determined at *p* < 0.05.

*Adh1*, *Adh4*, and *Cyp2e1* gene expression was measured in the liver of young adult HIV-1Tg and F344 normal rats treated with 0% EtOH (water control), 8% EtOH, or 52% EtOH in a 3-day binge regimen (total dose of 2.0 g/kg/d), using a custom-designed 48-gene PCR array. The fold change was calculated using the ΔΔCT method relative to the 0% EtOH group (*n* = 3 to 4 rats for each group) for each strain (F344 or HIV-1Tg).

Conversely, in the HIV-1Tg rats (Fig. 2*B*, Table 1), there were no EtOH concentration-dependent changes observed in the genes associated with EtOH metabolism. The 1.3- and 1.5-fold increases in *Adh1* and *Adh4*, respectively, in the 8% EtOH group, as well as the 1.6- and 1.3-fold respective increases in the 52% EtOH group, were not statistically significant nor was there any EtOH concentration-dependent pattern noted. Similarly, *Cy2pe1* expression was not significantly altered in response to EtOH concentration in either the 8% EtOH or 52% EtOH group.

**Fig. 3:**
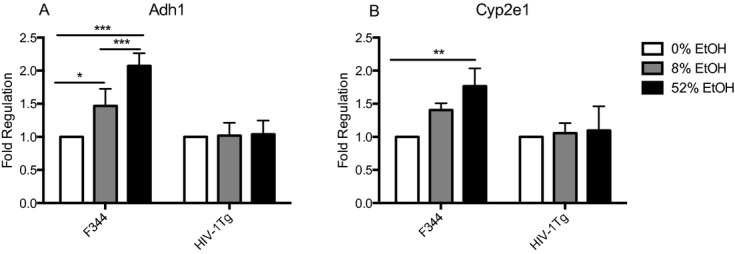
Ethanol (EtOH) concentration-dependent expression of EtOH metabolism-related genes, *Adh1* and *Cyp2e1*, in the liver of HIV-1Tg rats using real-time PCR analysis. *Adh1* and *Cyp2e1* expression was measured in the liver of young adult HIV-1Tg and F344 normal rats treated with 0% EtOH (water control), 8% EtOH, or 52% EtOH in a 3-day binge regimen (total dose of 2.0 g/kg/d), using real-time PCR. The fold change was calculated using the ΔΔCT method relative to the 0% EtOH group. Values represent the mean ± SD (*n* = 3 to 4 rats for each group). *p < 0.05, **p < 0.01, ***p < 0.001.

Using real-time PCR assay as a confirmatory method, we measured the gene expression of *Adh1* and *Cyp2e1* in response to a 3-day binge treatment with 0% (control), 8% EtOH, or 52% EtOH in the liver of HIV-1Tg and F344 normal rats (Fig. 3). In the F344 rats, both *Adh1* (Fig. 3*A*) and *Cyp2e1* (Fig. 3*B*) gene expression increased in an EtOH concentration-dependent manner. The fold increase, in comparison with the control group, was significantly higher in the 52% EtOH group (2.1-fold for *Adh1*; 1.8-fold for *Cyp2e1*) than the 8% EtOH group (1.5-fold for *Adh1*; 1.4-fold for *Cyp2e1*). However, in the HIV-1Tg rats, no EtOH concentration-dependent changes were observed in either *Adh1* or *Cyp2e1* gene expression.

### EtOH Concentration-Dependent Changes in Gene Expression in the Spleen of HIV-1Tg Rats Following a 3-Day Binge Treatment with 8% EtOH Versus 52% EtOH

**Fig. 4:**
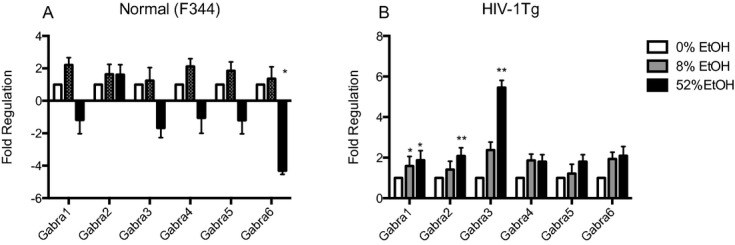
Ethanol (EtOH) concentration-dependent expression of GABA_A_ receptor genes in the spleen of HIV-1Tg rats using PCR array analysis. GABA_A_ receptor (*Gabra 1 to 6*) gene expression was measured in the spleen of young adult HIV-1Tg and F344 normal rats treated with 0% (water control), 8% EtOH, or 52% EtOH in a 3-day binge regimen (total dose of 2.0 g/kg/d), using a custom-designed 48-gene PCR array. The fold change was calculated using the ΔΔCT method relative to the 0% EtOH group. Values represent the mean ± SD (*n* = 3 to 4 rats for each group). *p < 0.05, **p < 0.01, ***p < 0.001.

There were significant EtOH concentration-dependent changes, particularly in the expression of neurotransmitter receptor genes, in the spleen of HIV-1Tg rats in comparison with F344 rats (Figs 6, Table 2). In the HIV-1Tg rats, the GABA receptor genes, particularly *Gabra 1*, *2*, and *3*, were significantly increased in the 52% EtOH group (1.9-, 2.1-, and 5.5-fold, respectively), but not in the 8% EtOH (1.6-, 1.4-, and 2.4-fold, respectively), compared with the 0% control group (Fig. 4*B*, Table 2). For *Gabra 4*, *5*, and *6*, there was increased expression compared with the control in both the 8% EtOH (1.8-, 1.2-, and 1.9-fold, respectively) and the 52% EtOH (1.8-, 1.8-, and 2.1-fold, respectively) groups, although the changes were not statistically significant.

**Table 2:** Ethanol (EtOH) Concentration-Dependent Expression of Neurotransmitter Receptor Genes in the Spleen of HIV-1Tg Rats

	Normal (F344) rats: up-down regulation				HIV-1Tg rats: up-down regulation			
		
Spleen	8% EtOH		52% EtOH		8% EtOH		52% EtOH	
				
Gene symbol	Fold regulation	*p*-Value	Fold regulation	*p*-Value	Fold regulation	*p*-Value	Fold regulation	*p*-Value
*Gabra1*	2.2125	0.325	−1.1788	0.644144	1.5898	0.035334	1.8752	0.026759
*Gabra2*	1.6383	0.963101	1.6128	0.866792	1.4158	0.455336	2.0846	0.009679
*Gabra3*	1.2467	0.485339	−1.6778	0.227888	2.3792	0.259119	5.4542	0.009679
*Gabra4*	2.1235	0.185104	−1.0529	0.547259	1.8617	0.281836	1.8027	0.346848
*Gabra5*	1.8577	0.278528	−1.2036	0.337454	1.2171	0.393527	1.8027	0.132689
*Gabra6*	1.3617	0.372891	−4.3087	0.014536	1.9397	0.181173	2.0936	0.098364
*Grm1*	2.1235	0.185104	−1.0529	0.547259	1.3789	0.328842	1.8027	0.009679
*Grm2*	2.1235	0.185104	−1.0529	0.547259	1.2171	0.393527	1.8027	0.009679
*Grm5*	2.0689	0.145467	−1.5056	0.434396	1.2029	0.639434	1.5163	0.253526
*Drd1a*	9.4615	0.161875	1.9185	0.18737	1.2256	0.397125	1.9516	0.003872
*Drd2*	−1.455	0.726711	−2.4636	0.036113	2.3164	0.320178	2.636	0.018461
*Drd3*	1.678	0.425532	−2.0507	0.071218	−1.3218	0.667884	2.1714	0.035732
*Drd4*	3.2467	0.137367	3.9139	0.014791	−1.1266	0.376632	−1.1026	0.436736
*Drd5*	2.0365	0.220021	−1.0607	0.52967	−1.1398	0.950395	1.7879	0.326432

Significance determined at *p* < 0.05.

Neurotransmitter receptor (GABA_A_ receptor [*Gabra 1 to 6*], metabotropic glutamate receptor [*Grm 1, 2*, and *5*], and dopamine receptors [*Drd1a, 2 to 5*]) gene expression was measured in the spleen of young adult HIV-1Tg and F344 normal rats treated with 0% (water control), 8% EtOH, or 52% EtOH in a 3-day binge regimen (total dose of 2.0 g/kg/d), using a custom-designed 48-gene PCR array. The fold change was calculated using the ΔΔCT method relative to the 0% EtOH group (*n* = 3 to 4 rats for each group) for each strain (F344 or HIV-1Tg).

**Fig. 5:**
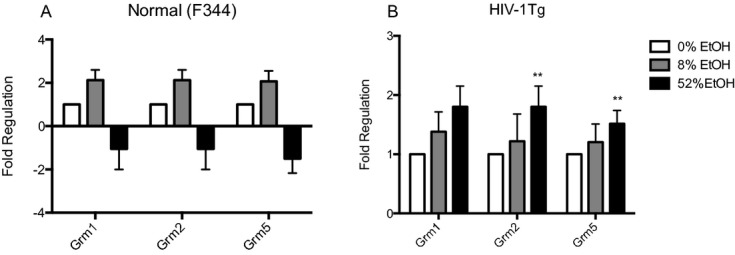
Ethanol (EtOH) concentration-dependent expression of metabotropic glutamate (mGlu) receptor genes in the spleen of HIV-1Tg rats using PCR array analysis. mGlu receptor (*Grm 1*, *2*, and *5*) gene expression was measured in the spleen of young adult HIV-1Tg and F344 normal rats treated with 0% EtOH, 8% EtOH, or 52% EtOH in a 3-day binge regimen (total dose of 2.0 g/kg/d), using a custom-designed 48-gene PCR array. The fold change was calculated using the ΔΔCT method relative to the 0% EtOH group. Values represent the mean ± SD (*n* = 3 to 4 rats for each group). *p < 0.05, **p < 0.01, ***p < 0.001.

**Fig. 6:**
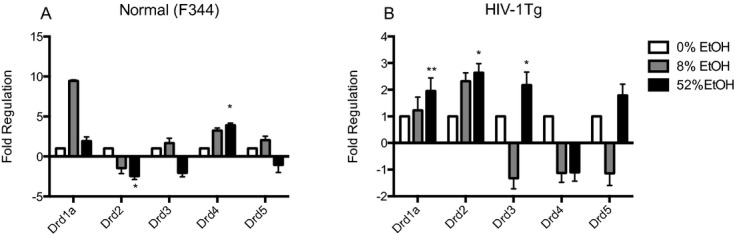
Ethanol (EtOH) concentration-dependent expression of dopamine receptor (DRD) genes in the spleen of HIV-1Tg rats using PCR array analysis. DRD (*Drd1a, 2 to 5*) gene expression was measured in the spleen of young adult HIV-1Tg and F344 normal rats treated with 0% EtOH (water control), 8% EtOH, or 52% EtOH in a 3-day binge regimen (total dose of 2.0 g/kg/d), using a custom-designed 48-gene PCR array. The fold change was calculated using the ΔΔCT method relative to the 0% EtOH group. Values represent the mean ± SD (*n* = 3 to 4 rats for each group). *p < 0.05, **p < 0.01, ***p < 0.001.

In the 8% EtOH group of F344 normal rats (Fig. 4*A*, Table 2), GABA receptor gene expression was slightly increased (2.2-fold for *Gabra1*; 1.6-fold for *Gabra2*; 1.2-fold for *Gabra3*; 2.1-fold for *Gabra4*; 1.9-fold for *Gabra5*; and 1.3-fold for *Gabra6*) compared with control, but the increases were not statistically significant. Conversely, in the 52% EtOH group of F344 normal rats, GABA receptor gene expression was decreased compared with control, except for *Gabra2* (1.6-fold increase), and the decrease in expression was statistically significant for only *Gabra6*.

The metabotropic glutamate (mGlu) receptor genes, *Grm1*, *Grm2*, and *Grm3*, were significantly increased in the 52% EtOH HIV-1Tg rats (1.8-, 1.8-, and 1.5-fold, respectively), but not in the 8% EtOH group (1.3-fold for *Grm1*; 1.2-fold for *Grm2*; and 1.2-fold for *Grm3*) in comparison with the control (Fig. 5*B*, Table 2). In the F344 rats (Fig. 5*A*, Table 2), mGlu receptor gene expression was decreased in the 52% EtOH group (−1.1-fold for *Grm1*; −1.1-fold for *Grm2*; and −1.5-fold for *Grm3*), and increased in the 8% EtOH group (2.1-, 2.1-, and 2.1-fold, respectively) compared with the control, although the changes were not statistically significant.

Expression of the dopamine receptor (DRD) genes was significantly increased in the 52% EtOH group (2.0-, 2.6-, 2.2-, and 1.8-fold for *Drd1a*, *Drd2*, *Drd3*, and *Drd5*, respectively), with the exception of *Drd4*, which was decreased 1.1-fold compared with the control in the HIV-1Tg rats (Fig. 6*B*, Table 2). There was no significant change observed in the 8% EtOH group. In the F344 rats (Fig. 6*A*, Table 2), there was no EtOH concentration-dependent pattern of change in DRD gene expression observed in the 52% EtOH group. Some receptor subtypes were decreased (−2.5-fold for *Drd2*; −2.1-fold for *Drd3*; and −1.1-fold for *Drd5*), whereas others were slightly increased (1.9-fold for *Drd1a*; 4.0-fold for *Drd4*) when compared to the control. Similarly, in the 8% EtOH group, *Drd2* was slightly decreased (−1.5-fold), whereas the other DRD subtypes were increased to various degrees (9.5-fold for *Drd1a*; 1.6-fold for *Drd3*; 3.2-fold for *Drd4*; and 2.0-fold for *Drd5*), although none of the changes were statistically significant.

**Fig. 7:**
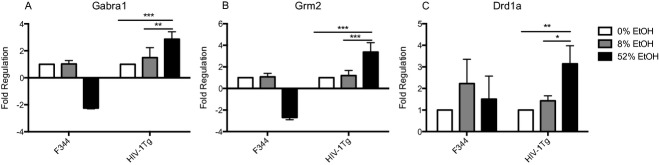
Ethanol (EtOH) concentration-dependent expression of neurotransmitter receptors genes, *Gabra1*, *Grm2*, and *Drd1a*, in the spleen of HIV-1Tg rats using real-time PCR analysis. *Gabra1*, *Grm2*, and *Drd1a* expression was measured in the liver of young adult HIV-1Tg and F344 normal rats treated with 0% EtOH (water control), 8% EtOH, or 52% EtOH in a 3-day binge regimen (total dose of 2.0 g/kg/d), using real-time PCR. The fold change was calculated using the ΔΔCT method relative to the 0% EtOH group. Values represent the mean ± SD (*n* = 3 to 4 rats for each group). *p < 0.05, **p < 0.01, ***p < 0.001.

To confirm our observations, we measured the gene expression of 1 representative gene (*Gabra1*, *Grm2*, and *Drd1a*) from each of the 3 neurotransmitter receptor families (GABA, mGlu, and dopamine; Fig. 7). As observed in our PCR array, no significant EtOH concentration-dependent differences were observed in the F344 rats. For all 3 genes (*Gabra1* [Fig. 7*A*], *Grm2* [Fig. 7*B*], and *Drd1a* [Fig. 7*C*]), the 8% EtOH group had a higher fold increase (1-fold for *Gabra1*; 1-fold for *Grm2*; 2.2-fold for *Drd1a*) than the 52% EtOH group (−2.2-fold for *Gabra1*; −2.7-fold for *Grm2*; 1.5-fold for *Drd1a*), when compared to control, although the changes were not statistically significant.

In the HIV-1Tg rats, significant EtOH concentration-dependent changes were observed. For all 3 genes, the 52% EtOH group had a significantly higher fold increase (2.9-fold for *Gabra1*; 3.4-fold for *Grm2*; 3.1-fold for *Drd1a*) than the 8% EtOH group (1.5-fold for *Gabra1*; 1.2-fold for *Grm2*; 1.4-fold for *Drd1a*).

## Discussion

The incidence of alcohol abuse in the HIV-1 infected population is significantly high, making it a serious concern for disease treatment and management. Antiretroviral medications, such as HAART, inhibit or block the replication part of the HIV-1 life cycle, but the virus cannot be completely eliminated (Guihot et al., 2011; Volberding and Deeks, 2010). Therefore, HIV-1 patients on antiretroviral therapy need to strictly adhere to their medication regimen or effectiveness is reduced (Volberding and Deeks, 2010).

Alcohol use and abuse have been found to reduce adherence to medication in the HIV-1-infected patients (Chander, 2011; Lucas et al., 2002; Samet et al., 1998; Wagner et al., 2001). Although HAART can control viral replication, other viral proteins, such as Tat, are still present, and these viral proteins have been found to enhance the deleterious effects of alcohol (Flora et al., 2005; Lawson et al., 2011; Mayne et al., 2000). Therefore, it is important to investigate whether alcoholic beverages are metabolized differently during the course of HIV-1 infection.

In this study, we found that there were differences in the BEC levels (Fig. 1) in HIV-1Tg rats given a 3-day binge treatment with high (52%) versus low (8%) EtOH concentration solutions compared with F344 normal rats, although the total amount of EtOH for each concentration was identical (2 g/kg/d for 3 days). The F344 normal rats given 8% EtOH had higher BEC levels 2 hours after the final treatment compared with the 52% EtOH group. Conversely, HIV-1Tg rats given 52% EtOH had significantly higher BEC levels 2 hours after the final treatment than the 8% EtOH group, indicating that there are differences in EtOH metabolism and processing in the HIV-1Tg rats compared with normal rats. We previously demonstrated that there are significant differences in BEC between a 52% EtOH and 20% EtOH binge treatment at 90 minutes posttreatment in F344 rats (Sarkar et al., 2013). We also found that only high concentrations of EtOH (>32%) are able to activate the neurons responsible for vasopressin production in the hypothalamus, thereby inducing dehydration (Chang et al., 1995; Sarkar et al., 2013). This could, in part, explain the EtOH concentration-dependent differences that we observed in this study. Another possible explanation for such EtOH concentration-dependent differences in BEC in the HIV-1Tg rats could also be differential expression of EtOH metabolism-related genes.

Using a custom-designed PCR array to examine expression of 48 genes specifically modulated by EtOH, we found that, in the liver of the F344 normal rats, *Adh1, Adh4*, and *Cyp2e1* were increased in an EtOH concentration-dependent manner, with a significant increase occurring after 52% EtOH compared with the control (Fig. 2*A*). To confirm our observations, we also used real-time PCR assay and found similar gene expression changes (Fig. 3). These data agree with previous observations that both *Adh4* and *Cyp2e1* have high activity levels and are more active at high EtOH concentrations (Edenberg, 2007; Zakhari, 2006), and indicate that high EtOH concentration solutions are metabolized faster owing to the increased expression of EtOH metabolism-related genes, thereby resulting in lower BEC levels in the 52% EtOH group compared with the 8% EtOH group.

However, in the HIV-1Tg rats (Fig. 2*B*), there were no significant EtOH concentration-dependent changes in the genes associated with EtOH metabolism. Haorah and colleagues (2004) reported that *Cyp2e1* activity was lower in monocyte-derived macrophages in the presence of HIV-1 infection, suggesting that the activity of EtOH metabolism-related genes, particularly the high kinetic activity enzymes such as *Adh4* and *Cyp2e1*, may be lower during the course of HIV-1 infection (Edenberg, 2007; Zakhari, 2006). The lower activity of these EtOH metabolism-related genes in the HIV-1Tg rats given a high EtOH concentration solution could be 1 reason for the elevated BEC levels we observed in the HIV-1Tg rats.

The spleen is an important organ responsible for destroying aged red blood cells (Mebius and Kraal, 2005). The spleen also produces antibodies and houses more than half of the body's monocytes, which, in the event of tissue damage, differentiate into macrophages that travel to the site of injury (Swirski et al., 2009). During HIV-1 infection, the spleen is affected by the virus and other opportunistic infections causing impairment in splenic macrophage activity (Falk and Stutte, 1990; Klatt and Meyer, 1987), thereby suppressing immune function. Alcohol is an immunosuppressant (Baum et al., 2010) that could further suppress splenic immune function. The spleen is also a highly innervated lymph organ, thus providing a platform for neuro-immune connections. The splenic monocytes and macrophages house neurotransmitter receptors and, hence, are capable of neuromodulatory effects (Straub et al., 2000). Neurotransmitter activity is mediated through the expression of their receptors (Valenzuela, 1997). Alcohol can modulate neurotransmitter activity by changing the homeostasis between excitatory and inhibitory pathways (Valenzuela, 1997). Short-term alcohol drinking, such as binge drinking, can affect inhibitory neurotransmitter functions (Pohorecky, 1977).

In this study, we found that, in the spleen of HIV-1Tg rats given high concentrations of EtOH (52%), there were significant increases in the expression of neurotransmitter receptor genes, including GABA_A_ (*Gabra 1*, *2*, and *3*), mGlu (*Grm 1* and *2*), and DRD (*Drd1a*, *2* and *3*) compared with the normal F344 rats using both PCR array (Figs 6) and real-time PCR methods (Fig. 7). Conversely, GABA_A_ receptor expression was decreased in the normal rats given a 52% EtOH solution, and confirmed our previous findings that GABA_A_ receptor expression is decreased in adolescent F344 rats given 52% EtOH (Liu et al., 2011). This decrease in GABA_A_ receptor expression in the spleen of the F344 normal rats in our study could alter the effects of the GABA_A_ inhibitory neurotransmitter, resulting in an imbalance between inhibitory and excitatory pathways.

The HIV-1 viral protein, Tat, can induce secretion of many neurotransmitters, including glutamate and dopamine (Neri et al., 2007; Scheller et al., 2010). There is an EtOH concentration-dependent increase in Tat expression in the spleen of HIV-1Tg rats (Sarkar et al., 2013), indicating that the spleen is susceptible to the damaging effects of EtOH, particularly at high concentrations (52%). Thus, in the HIV-1Tg rats, there could be synergistic effects of HIV-1 Tat and EtOH that causes increased neurotransmitter receptor expression and alters receptor pathway functions.

In summary, our findings provide new information strongly suggesting that there are EtOH concentration-dependent effects of acute binge drinking that are differentially manifested in the presence of HIV-1 infection.
